# Homeopathic College

**Published:** 1860-10

**Authors:** 


*Homeopathic College.*—New York has distinguished itself by instituting an incorporated Homeopathic College, and we are a little surprised to see some very respectable names as corporators. Its faculty are men unknown outside of the city, or to science,—boasting the title of M. D., which, if worth anything, would utterly refute all scientific claims by their Hannemhannic theory. According to close observation, it is gradually wearing out the world over. Its only well known advocate, Henderson, is, by grace, in a respectable position professionally; but by justice, a dead man in the scientific world. B.

